# Clinicopathological findings in a case series of extrathoracic solitary fibrous tumors of soft tissues

**DOI:** 10.1186/1471-2482-6-10

**Published:** 2006-07-06

**Authors:** Adrien Daigeler, Marcus Lehnhardt, Stefan Langer, Lars Steinstraesser, Hans-Ulrich Steinau, Thomas Mentzel, Cornelius Kuhnen

**Affiliations:** 1 Department of Plastic Surgery, Burn Center, Hand Center, Sarcoma Reference Center, BG-University-Hospital "Bergmannsheil", Ruhr-University Bochum, Germany; 2 Dermatohistopathologisches Gemeinschaftslabor, Friedrichshafen, Germany; 3 Institute of Pathology, BG-Hospital "Bergmannsheil", Ruhr-University, Bochum, Germany

## Abstract

**Background:**

Solitary fibrous tumors (SFT) represent a rare entity of soft tissue tumors. Previously considered being of serosal origin and solely limited to the pleural cavity the tumor has been described in other locations, most particularly the head and neck. Extrathoracic SFT in the soft tissues of the trunk and the extremities are very rare. Nine cases of this rare tumor entity are described in the course of this article with respect to clinicopathological data, follow-up and treatment results.

**Methods:**

Data were obtained from patients' records, phone calls to the patients' general practitioners, and clinical follow-up examination, including chest X-ray, abdominal ultrasound, and MRI or computed tomography.

**Results:**

There were 6 females and 3 males, whose age at time of diagnosis ranged from 32 to 92 years (mean: 62.2 years). The documented tumors' size was 4.5 to 10 cm (mean: 7 cm). All tumors were located in deep soft tissues, 3 of them epifascial, 2 subfascial, 4 intramuscular. Four tumors were found at the extremities, one each at the flank, in the neck, at the shoulder, in the gluteal region, and in the deep groin. Two out of 9 cases were diagnosed as atypical or malignant variant of ESFT. Complete resection was performed in all cases. Follow-up time ranged from 1 to 71 months. One of the above.mentioned patients with atypical ESFT suffered from local relapse and metastatic disease; the remaining 8 patients were free of disease.

**Conclusion:**

ESFT usually behave as benign soft tissue tumors, although malignant variants with more aggressive local behaviour (local relapse) and metastasis may occur. The risk of local recurrence and metastasis correlates to tumor size and histological status of surgical resection margins and may reach up to 10% even in so-called "benign" tumors. Tumor specimens should be evaluated by experienced soft tissue pathologists. The treatment of choice is complete resection followed by extended follow-up surveillance.

## Background

Solitary fibrous tumors (SFT) – previously known as benign fibrous mesotheliomas – were considered being of serosal origin and being exclusively located in the thoracic cavity as pleural fibrous tumors. More recently, however, SFT have been reported in many extrapleural locations such as the head and neck region; in particular, the meninges, the orbita, the nasal and oral cavity, the salivary glands, and the visceral organs, the retroperitoneum, and the pelvic space. Extrapleural solitary fibrous tumors (ESFT), especially those at the extremities, still represent a rare entity of soft tissue tumors. In a previous study extrapleural solitary fibrous tumors (ESFT) counted for 0.6% of all soft tissue tumors, sent in for analysis [1]. In fact, by taking into account the bias caused by the fact that rare tumor specimens accumulate at specialised institutions, the general proportion might be much smaller. Usually the tumor is of benign behaviour (showing no local recurrence or metastasis), but malignant variants have also been described and local recurrence as well as metastasis may occur depending on the initial tumor size and the histological status of the surgical resection margins [1-18]. We report 9 cases of ESFT including 4 tumors localized at the extremities with clinicopathological, immunohistochemical and follow-up data.

## Methods

From January 1999 to May 2005, nine patients were diagnosed with extrapleural SFT at our institutions. Data were obtained from patients records and phone calls to the patients and their general practitioners.

### Pathological examination

Preoperative incisional biopsy specimens were obtained in 4 of 9 cases. The resection specimens were examined regarding tumor size, exact location, extension to adjacent soft tissues, and cut surface. A macroscopic photograph of cut surface was obtained in selected cases.

Histopathological evaluation was performed by experienced soft tissue pathologists.

The criteria for histopathological diagnosis included the widely accepted characteristic features of SFT (WHO 2003: patternless growth pattern, composed of round to spindle-shaped fibroblastic cells set in a collagenous matrix, hemangiopericytoma-like vasculature pattern with often hyalinized thickened vessel walls and characteristic immunohistochemical findings). Light microscopical analysis was based on H&E stained slides. Immunohistochemical analysis included the following antibodies (table 1): vimentin, CD 34, CD 99, BCL-2, keratin AE1/AE3, keratin MNF116, EMA, S-100-protein and Ki-67 (antibody MIB-1).

Follow-up data were available for all patients and consisted of clinical examination, chest X-ray, abdominal ultrasound and computed tomography or MRI of the tumor site. Follow-up time ranged from 1 to 71 months (mean: 26.5 months).

## Results

**Figure 1:**
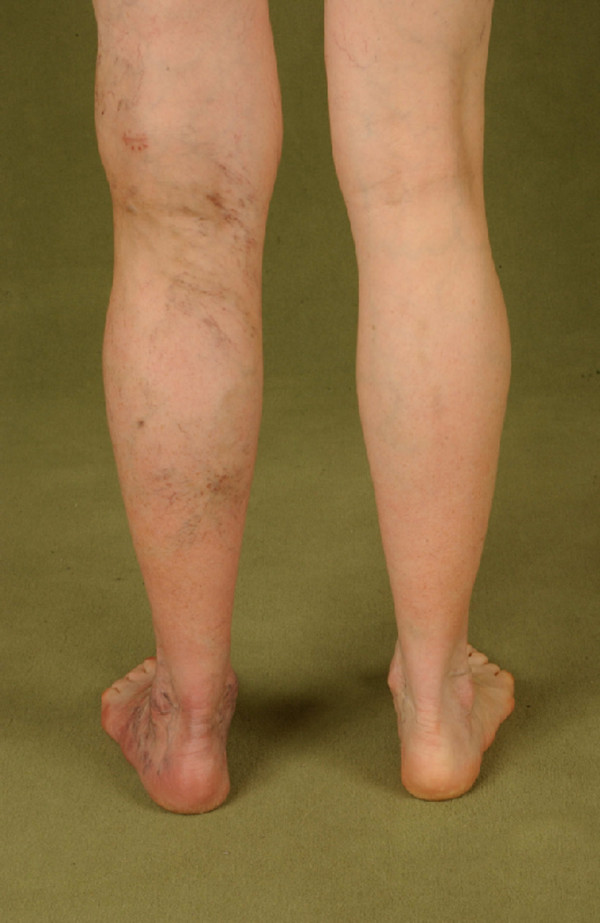
**clinical aspect of SFT**. Clinical aspect of a solitary fibrous tumor of the lower left leg: swelling with soft tissue mass (same patient as in fig. 3).

**Figure 2:**
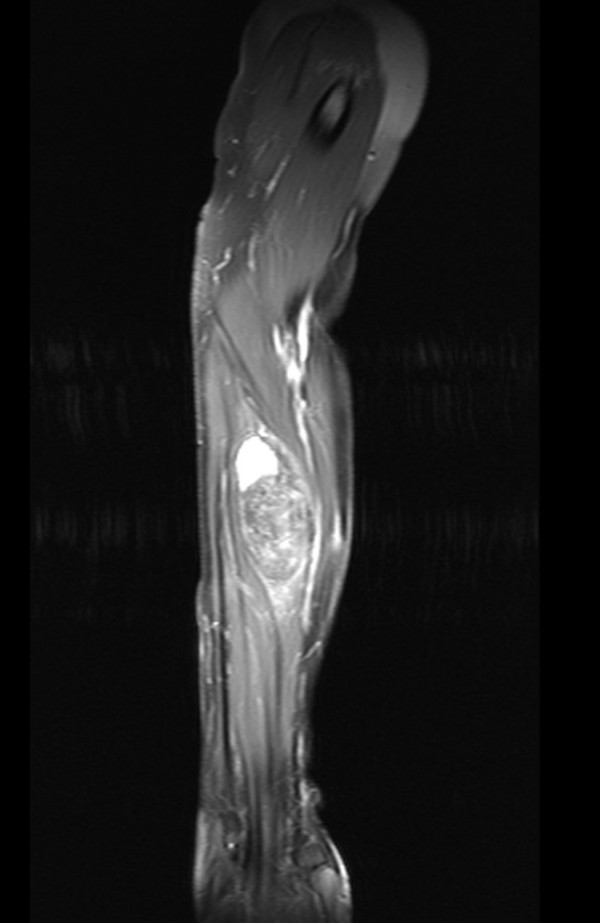
**MRI of SFT**. MRI of a solitary fibrous tumor depicting a relatively well circumscribed soft tissue tumor in the forearm.

The patients included 6 women and 3 men. Their age at time of diagnosis ranged from 32 to 92 years and averaged 62.2 years. Tumors were painless or became symptomatic by their mass effect, causing localized pain, or as in one patient, hypaesthesia. There were no other (especially no generalized) symptoms. The tumors existed 5 months to 5 years before being diagnosed. All tumors were located in deep soft tissues, 3 of them epifascial, 2 subfascial, 4 intramuscular. Four tumors were located at the extremities (thigh, fossa poplitea (Fig. 1), 2× lower arm (Fig. 2)) and one each in the flank, the neck, at the shoulder, in the deep groin, and in the gluteal region. The diameters of the tumors ranged from 4.5 to 10 cm (mean: 7 cm). In 7 patients, primary resection could be performed with free surgical margins; four of them were primarily incisional biopsied. In one patient, treated externally, the resection had to be considered a R1-resection due to an intraoperative tumor incision with possible contamination of the situs. Whether or not this will influence the long term outcome cannot yet be determined because of a follow-up time of 1 month. Another patient with unfavourable clinical course (Patient 5) presented at our institution after pre-treatment at another institution 2 years before consisting of incomplete primary resection and secondary resection then with free margins, followed by 60 Gy of radiation. This patient showed extensive local recurrence and an amputation at upper arm level had to be performed. Five months later, the patient presented again with two new metastases: one in the right axilla and another one epifascial at the right subscapular region which were resected with free surgical margins. At the time of this study all other patients had no evidence (NED) of disease. The detailed clinical findings are summarized in table 2.

### Pathological findings

**Figure 3:**
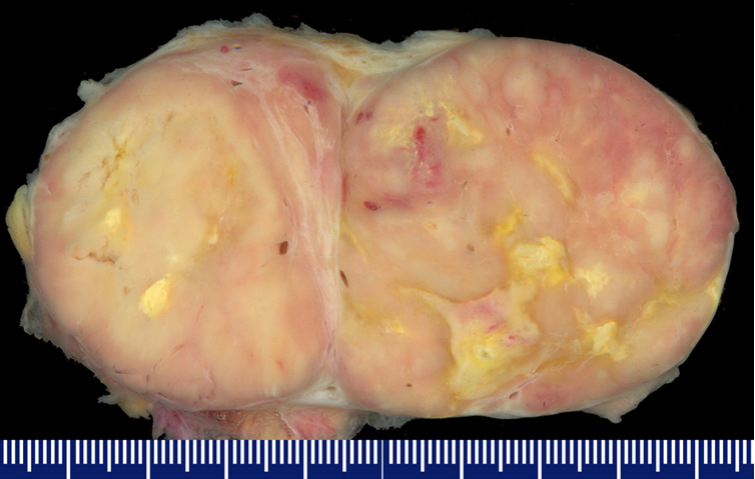
**Macroscopic aspect of SFT**. Macroscopic aspect of a solitary fibrous tumor: cut surface of a well circumscribed tumor; white to tan coloured cut surface with some deeply yellow necrotic areas (atypical/malignant solitary fibrous tumor, case 8).

The tumors appeared as solid, mostly well-circumscribed and smooth to firm soft tissue masses. The cut surface appeared white to brown-yellow. Considerable tumor necrosis was evident in case 5 and 8 (Fig. 3).

**Figure 4:**
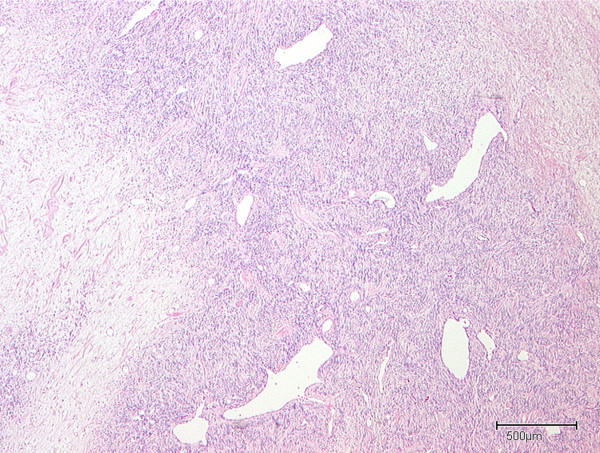
**Microscopic aspect of SFT (a)**. Microscopic aspect of a solitary fibrous tumor: "Patternless" growth pattern with cellular (right half) and some myxoid (left half) areas, hemangiopericytoma-like wide blood vessels.

**Figure 5:**
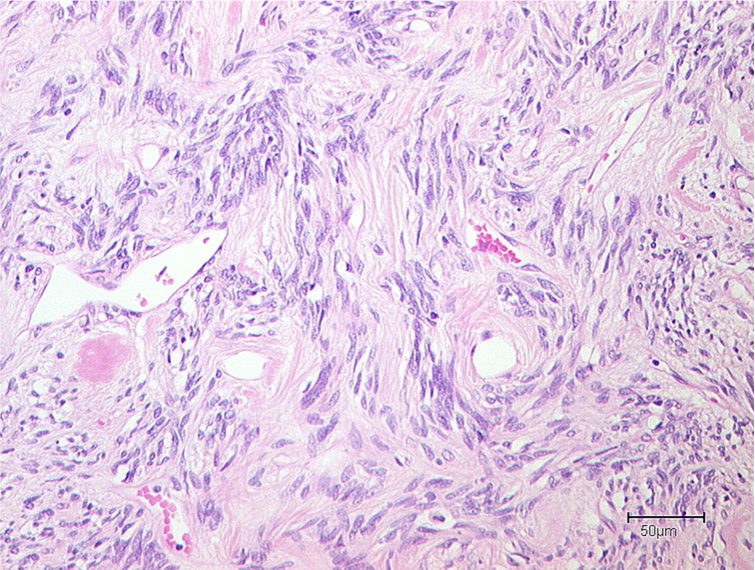
**Microscopic aspect of SFT (b)**. Microscopic aspect of a solitary fibrous tumor, higher magnification: fibroblastic, partly "neural-like" tumor cells lying in a fibrous, partly hyalinized matrix, hemangiopericytoma-like blood vessels.

The tumors were composed of fibroblastic appearing cells, usually showing a characteristic "patternless" growth pattern with small fascicular areas (Fig. 4). The vessels adopted a hemangiopericytoma-like growth pattern consisting of elongated and dilated vessels with thickened, often hyalinized walls (Fig. 5). The tumor matrix included variable amounts of partly hyalinzed collagen bundles. Truly infiltrative growth patterns could not be detected, although vascular infiltration was evident in one case (Patient 5). The tumor cells were characterized by "ovally" to spindle-shaped nuclei, sometimes resembling neural cytologic features (i.e., wavy nuclei). The mitotic rate in morphologically benign SFTs was less than 4/10 HPF (high power field: objective × 40). Two lesions were diagnosed as atypical variant of ESFT due to a markedly increased cellularity, cellular atypia (nuclear pleomorphism, nuclear hyperchromasia), increased mitotic index and tumor necrosis (Patient 5+8).

All neoplasms stained variably positive for CD 34, CD 99, BCL-2 and vimentin. An additional expression of smooth muscle actin was seen in 3 of 5, muscle specific actin in 1 of 3 and expression of desmin could be detected in 2 of 4 examined cases. No positive keratin and S100 immunoreaction was noted, whereas EMA could be detected focally in 2 cases. The proliferation rate ranged from 1 to 10%. A detailed summary of the histopathological findings is given in table 3.

## Discussion

SFT represents a distinct entity within the wide range of soft tissue tumors. Its cellular origin is believed to be fibroblastic in type. Most cases of formerly diagnosed "hemangiopericytomas" seemingly share essential features with SFT and may indeed represent true SFT. According to overlapping histological criteria between SFT and hemangiopericytoma, SFT represents a wider range of neoplasias (probably in fact including the former "favourite" diagnosis of hemangiopericytoma) which should best be regarded as a "waste basket" and be considered a mere diagnosis of exclusion. The lipomatous variant of SFT ("lipomatous hemangiopericytoma") includes a mature fat component intermingling with typical areas of SFT. Rare myxoid SFT may cause considerable problems in differential diagnosis to more aggressive soft tissue neoplasms or soft tissue tumors of another differentiation. Branching and ectatic blood vessels typical for SFT known as hemangiopericytoma-like vessels may be a feature of several other predominantly malignant soft tissue tumors (e.g. synovial sarcomas or malignant peripheral nerve sheath tumors), implicating that a wider range of other soft tissue neoplasias has to be considered in the histopathological differential diagnosis of SFT. The so-called "patternless pattern" or the combination of different histological patterns such as storiform, fascicular, neuraltype, diffuse sclerosing, and heringbone growth pattern may lead to a wrong diagnosis [19]. Therefore, it is evident that an experienced soft tissue pathologist should evaluate the specimens. For differential diagnosis, so-called hemangiopericytoma, synovial sarcoma, dermatofibrosarcoma protuberans, leiomyosarcoma, malignant peripheral nerve sheath tumor, and liposarcoma, should be taken into consideration. Positron emission tomography (PET) may be helpful to distinguish between a malignant and a benign variant of the tumor [20-22], but the gold standard for diagnosis remains incisional biopsy.

Extrathoracic solitary fibrous tumors (ESFT) by now have been reported at almost every anatomic location, but reports of tumors at the extremities or intramuscular tumors as well as tumors with malignant clinical behaviour or atypical histologic features are rare [1-18].

Other series of ESFT's showed almost equal distribution of the incidence for male and female, with patients' ages ranging from the third to the eighth or ninth decade with a maximum in the sixth decade concurring with our data [3,7,8].

Some studies suggested a very low rate of recurrence and metastasis [8,11,23], whereas other authors indicated a possibly increased relapse rate with extended follow-up periods. In their studies Vallat-Decouveleare et al. and Gold et al. [3] in their studies found local recurrence in 4.3% and 6.7% and metastasis in 5.4% and 5.3%, respectively. Tumor relapse occurred after up to 168 months, but most of the metastasis or local recurrences were diagnosed within the first two years after initial treatment. Sites of distant metastasis were lung, liver, bones, mesentery, omentum, mediastinum and retroperitoneum with preference for lung and liver [1,3].

Vallat-Decouveleare suggested atypical histologic features, such as nuclear atypia, areas of increased cellularity, necrosis and 4 or more mitoses per 10 HPF as being predictive for clinical malignant behaviour of the tumor and found local or distant relapse in those cases in 80%, but also reported a case of clinically malignant behaviour of a histological benign appearing case. Recurrent tumor specimens showed a higher grade of atypia than the primary tumor but usually retained their immunohistochemical profile. Gold's data proposed to add the size of the primary tumor as well as the resection status to the predictive factors of clinical behaviour. Positive surgical resection margins and primary tumor sizes of more than 10 cm were positively correlated with unfavourable clinical outcome.

In our series, the only patient with recurrent disease and metastasis was primarily resected incompletely, underlining this suggestion. In the recurrent tumor specimen increased atypia was seen, but if this atypia had already been present in the primary tumor and therefore could be considered as of predictive value cannot be determined. The recurrence occurred 2 years and the metastasis 2 ½ years after primary resection and following radiation. Detailed histopathological information about the primary tumor could not be obtained.

Complete surgical resection is commonly accepted as treatment of choice for ESFT. Due to improved techniques in reconstructive surgery, even large lesions can usually be completely resected, preserving the limbs. Amputations should be limited to extended or recurrent tumors. Befitting the rareness of this entity reports of radiation therapy and chemotherapy of ESFT are anecdotal and so far, no significant benefit of adjuvant treatment has been reported. In some cases, especially malignant variants or incomplete resections with no further surgical option, it may although be used [1,3]. This concurs with the findings for intrathoracic SFT [24,25].

According to the late recurrence or metastasis, long follow-up periods (at least 15 years) should be maintained with closer follow-up during the first two years. In cooperative patients a life long follow-up may be recommended. Follow-up should include clinical examination as well as abdominal ultrasound and chest x-ray.

## Conclusion

Soft Tissue sarcoma specimens should be evaluated by experienced soft tissue pathologists for correct diagnosis of SFT and detection of atypia. SFT with atypical histologic features, such as nuclear atypia, areas of increased cellularity, necrosis and 4 or more mitoses per 10 HPF, tumor sizes of more than 10 cm and incomplete resection are positively correlated with local recurrence and metastatic disease. Therefore complete resection at an early stage should be the main purpose of surgical treatment. Follow-up should be maintained for 10 years.

## Competing interests

The author(s) declare that they have no competing interests.

## Authors' contributions

AD designed the study, obtained the patients' data and drafted the manuscript.

ML contributed to manuscript preparation and helped designing the study

SL contributed to evaluation of the data

LS contributed to obtaining the data

HS revised the manuscript, gave substantial intellectual input concerning evaluation of the data and gave final approval of the version to be published

TM contributed to manuscript preparation and histopathological evaluation

CK participated in the study design, histopathological evaluation, and manuscript preparation

All authors read and approved the final manuscript.

## Pre-publication history

The pre-publication history for this paper can be accessed here:



## Figures and Tables

**Table 1:** Antibodies

*Antibody*	*Source*	*Dilution*	*Clone*
CD 99	DAKO	1:25	12E7
CD 34	Immunotech	1:2000	QBEND10
BCL-2	DAKO	1:6	124
Vimentin	DAKO	1:8000	V9
EMA	DAKO	1:50	E29
Keratin MNF 116	DAKO	1:1600	MNF 116
Keratin AE1/AE3	DAKO	1:800	AE1/AE3
Ki67	DAKO	1:800	MiB1
S100	DCS	1:200	15E2E2

Detailed data about the antibodies used

**Table 2:** Clinical findings

*patient*	*gender*	*age at time of diagnosis *(*years*)	*localisation/depth of tumor*	*size of primary tumor *(*cm*)	*presentation status of tumor*	*symptoms on first presentation*	*pre-operative incisional biopsy*	*surgical margins in final procedure*	*local recurrence*	*metastasis*	*secondary procedures*	*status at last follow-up *(*months*)
*1*	male	40	neck/epifascial	10 × 8 × 10	primary	neck pain	no	negative	no	no	none	alive, NED, 21
*2*	male	56	shoulder/epifascial	4.5 × 4 × 2.5	primary	painless mass	no	negative	no	no	none	alive, NED, 56
*3*	female	85	m. vastus lateralis	4.8 × 3 × 2	primary	painless mass	yes	negative	no	no	none	alive, NED, 71
*4*	female	32	m. iliopsoas	4.5 × 3.3 × 2.1	primary	groin pain	no	negative	no	no	none	alive, NED, 38
*5*	male	57	lower arm/flexor muscles	no data	local recurrence	painless mass	no	negative	yes, after 2 years	yes, after 3 years	axillary dissection, amputation at upper arm level resection of two metastases	alive, NED, 6
*6*	female	59	lower arm/flexor muscles	6.2 × 3.5 × 4.2	primary	painless mass	yes	negative	no	no	none	alive, NED 12
*7*	female	92	flank/epifascial	10 × 10 × 9	primary	painless mass	yes	negative	no	no	none	alive, NED 28
*8*	female	52	fossa poplitea/subfascial	9.5 × 5.4 × 6.5	primary	pain in fossa poplitea, paraesthesia lateral lower leg	yes	negative	no	no	none	alive, NED 6
*9*	female	87	gluteal/subfascial	6.6 × 5.6 × 3.7	primary	Painless swelling	no	R1	no	no	none	Alive, NED 1

Summary of the patients' data (no evidence of disease: NED)

**Table 3:** Histological results

*patient*	*CD34 reactivity*	*CD99 reactivity*	*BCL2 reactivity*	*Vimentin *(*V9*) *reactivity*	*actin HHF35*	*smooth muscle actin*	*desmin*	*S100*	*keratin*	*MNF116*	*EMA*	*MiB1*	*necrosis*	*average mitoses/10 HPF*	*malignant component*	*additional tests*
*1*	focal positive	focal positive	positive	positive	-	-	-	-	-	-	-	-	absent	<4	absent	
*2*	focal positive	-	-	positive	-	focal positive	-	negative	negative	-	-	1%	absent	<4	absent	
*3*	positive	positive	-	positive	focal positive	focal positive	focal positive	negative	negative	-	-	5%	absent	<4		loss on chromosome 13q
*4*	focal positive	focal positive	focal positive	focal positive	-	focal positive	focal positive	-	negative	negative	-	1–2%	absent	<1	absent	
*5*	positive	positive	-	positive	-	-	-	negative	negative	-	negative	5–10%	present	10	present	HMB 45 negative
*6*	focal positive	positive	positive	positive	negative	negative	negative	negative	negative	negative	focal positive	<5%	absent	<4	absent	Keratin AE1/AE3 negative
*7*	positive	positive	positive	positive	-	-	-	-	-	-	focal positive	<1%	absent	<4	absent	
*8*	positive	positive	positive	positive	-	-	negative	negative	negative	negative	negative	5%	present	3	present	HMB 45 negative Keratin AE1/AE3 focal
*9*	positive	positive	positive	focal positive	negative	negative	-	-	-	-	-	5–10%	absent	<4	absent	

Summary of the histological results (-: not tested)
